# Introducing the refined gravity hypothesis of extreme sexual size dimorphism

**DOI:** 10.1186/1471-2148-10-236

**Published:** 2010-08-03

**Authors:** Guadalupe Corcobado, Miguel A Rodríguez-Gironés, Eva De Mas, Jordi Moya-Laraño

**Affiliations:** 1 Dpto. de Ecología Funcional y Evolutiva, Estación Experimental de Zonas Áridas, Consejo Superior de Investigaciones Científicas, Carretera de Sacramento, s/n. La Cañada de San Urbano, C.P. 04120. Almería, Spain; 2 Cantabric Institute of Biodiversity (ICAB). Dpto. de Organismos y Sistemas. Universidad de Oviedo, Catedrático Rodrigo Uría, s/n, 33006-Oviedo, Spain

## Abstract

**Background:**

Explanations for the evolution of female-biased, extreme Sexual Size Dimorphism (SSD), which has puzzled researchers since Darwin, are still controversial. Here we propose an extension of the Gravity Hypothesis (i.e., the GH, which postulates a climbing advantage for small males) that in conjunction with the fecundity hypothesis appears to have the most general power to explain the evolution of SSD in spiders so far. In this "Bridging GH" we propose that bridging locomotion (i.e., walking upside-down under own-made silk bridges) may be behind the evolution of extreme SSD. A biomechanical model shows that there is a physical constraint for large spiders to bridge. This should lead to a trade-off between other traits and dispersal in which bridging would favor smaller sizes and other selective forces (e.g. fecundity selection in females) would favor larger sizes. If bridging allows faster dispersal, small males would have a selective advantage by enjoying more mating opportunities. We predicted that both large males and females would show a lower propensity to bridge, and that SSD would be negatively correlated with sexual dimorphism in bridging propensity. To test these hypotheses we experimentally induced bridging in males and females of 13 species of spiders belonging to the two clades in which bridging locomotion has evolved independently and in which most of the cases of extreme SSD in spiders are found.

**Results:**

We found that 1) as the degree of SSD increased and females became larger, females tended to bridge less relative to males, and that 2) smaller males and females show a higher propensity to bridge.

**Conclusions:**

Physical constraints make bridging inefficient for large spiders. Thus, in species where bridging is a very common mode of locomotion, small males, by being more efficient at bridging, will be competitively superior and enjoy more mating opportunities. This "Bridging GH" helps to solve the controversial question of what keeps males small and also contributes to explain the wide range of SSD in spiders, as those spider species in which extreme SSD has not evolved but still live in tall vegetation, do not use bridging locomotion to disperse.

## Background

Sex differences in body size, or sexual size dimorphism (SSD), are widespread across the animal kingdom [1-4], and hypothetically reflect the different selective pressures acting on males and females [5,6]. Female-biased SSD (females larger than males) is the most common pattern, typical in invertebrates and ectothermic vertebrates. Even though fecundity selection favoring large females [1,7] is the most accepted explanation for female-biased SSD, two questions remain unsatisfactorily solved. First, what selective pressures act on males in order to keep them small [5,8] in spite of the usually high genetic correlation between the sexes? [9]; and second, what factors are responsible for the wide range of variation in SSD present in nature? [10].

SSD is widespread among spiders (Araneae). There is a general pattern of female-biased SSD which is highly variable among taxa [11,12]. Additionally, spiders are the only group of free-living terrestrial organisms in which extreme SSD - i.e., females twice as large as males [13]-, is common, and in which this pattern has evolved several times independently [13]. Most spider species exhibiting extreme SSD are found among Orbicularian spiders (especially in the families Tetragnathidae, Theridiidae and Araneidae) and the family Thomisidae within the RTA clade. However, some exceptional cases can be found in other taxa, including the Mygalomorphae [14]. Hormiga *et al*. [13] in a study based on 9 families of Orbicularian spiders (526 species) found that 24 out of 76 genera showed female-biased extreme SSD and that extreme SSD had been gained and lost several times across the phylogeny. Vollrath & Parker [15] found that in web-building spiders (7 families from the Orbiculariae clade plus the families Agelenidae and Atypidae - the latter belonging to the old Mygalomorphae clade-), 93 out of 159 species showed extreme SSD, and 17 out of the 20 species of sampled Thomisidae also showed extreme SSD. In contrast none of the 41 species from the 7 families: Pisauridae, Lycosidae, Salticidae, Philodromidae, Sparassidae, Clubionidae and Gnaphosidae, showed extreme SSD [15]. Thus, spiders, especially those from the Orbicularian clade and the family Thomisidae, constitute a perfect system to study the evolution of extreme SSD.

In spiders, the most accepted hypothesis explaining the actual pattern of SSD is the fecundity selection hypothesis, by which females increase in size due to selection imposed on fecundity [7,13,16-18]. Nevertheless, this hypothesis does not offer a solid explanation for the wide range of SSD found across spiders. In particular, fecundity selection does not explain why there are some species in which males grow almost as giant as females while in others males stay small while females grow gigantic. Additionally, there is a strong controversy about which selective pressures act on male size and thus on the adaptative significance of SSD [12]. In spiders, males and females share the same lifestyles until maturation. Following maturation males, which are generally the searching sex, change their lifestyle and start roaming, searching for females to mate with [[19], but see [20]]. As a consequence of this particular male lifestyle, many hypotheses have tried to explain the adaptive significance for the evolution of SSD in relation to male agility or the ability of males to find receptive females, which usually point to advantages of small body size [1,15,21-25]. However, a global and integrative explanation for the evolution of extreme SSD has not been achieved thus far [reviewed in [12]].

The gravity hypothesis (GH) [25] links the evolution of extreme SSD with the capacity of males to move on vertical surfaces. The GH predicts that smaller males should be favored because they climb faster and, as a result, these males would have an advantage either by scramble competition (more mating opportunities) or natural selection (escaping from predators). Although there is some controversy around the GH [26-30], it has been shown that the relationship between climbing speed and body mass is curvilinear with an optimal body mass for climbing [31]. Consequently, it has been suggested that extreme SSD has evolved only in those species that 1) live in high habitats and 2) in which females are larger than the optimal climbing mass.

However, in species living in high habitats spiders do not only walk or climb to disperse: they also bridge. Bridging is a very common means of aerial locomotion in spiders [32-34] that has nevertheless been relatively neglected in the literature [but see [35-40]]. To bridge, a spider releases a line of silk that the wind attaches to a distant plant, and after actively tensing the line by pulling it back with its legs, the spider crosses hanging upside-down from the line [38]. Different sources of evidence support the hypothesis that the morphology (leg length relative to body size) of modern spiders (Araneomorphae) that hang upside-down from their webs, has evolved to facilitate bridging, allowing spiders to swing as pendulums from their silk threads [38]. This finding suggests that bridging plays an important role in the life of some spider taxa [33], and that during evolutionary time morphological adaptations to bridging locomotion should be expected in spiders that live in high places [38]. Given that dispersal through bridging is very uncommon among very large individuals (GC and JML, *personal observations*), constraints on bridging could help to explain the evolution of SSD in spiders.

Morse & Fritz [35] hypothesized that heavy *Misumena vatia *females did not use bridging for long-distance dispersal because, due to the elasticity of silk, the sag of the fiber would bring the spiders down to the ground when they crossed their silk bridges. This hypothesis is confirmed by a biomechanical model showing that large spiders cannot use bridging as an efficient dispersal mechanism. Large spiders are limited to short bridging events or to bridging events that start high above the surface (Rodríguez-Gironés et al. *unpublished manuscript*). Spiders have up to 5 different types of silk glands which spin fibrous silks characterized by different mechanical properties and linked to distinct ecological functions [42-44]. The kind of silk used to bridge is spun in the minor ampullate glands [33,34]. Some remarkable properties of this kind of silk are the thin diameter of its fibers, their low strength and their high extensibility [44]. Although spiders have some control on the diameter of their minor ampullate silk fibers [45], as well as the diameter of other types of lines [eg. [46-50]], phenotypic plasticity in thread diameter is severely limited [51], possibly constraining the ability of large spiders to bridge. Thus, while giant female spiders perform short bridging events during web building [33], large adult web-building spiders move less often and shorter distances than juveniles [52]. This could be due to the fact that large spiders are not able to bridge long distances efficiently (Rodríguez-Gironés et al. *unpublished manuscript*).

It is easy to see how a small size could be favored in habitats in which bridging is the most frequent mode of locomotion. If large spiders forego bridging, they will have to walk along leaves, twigs and branches, climbing down to the ground, walking towards their target and climbing up again. Other than the extra time and energy expended by climbing up and down, this implies an increase in the length of their trajectory. In addition, once spiders are morphologically adapted to bridge, this would be particularly disadvantageous, as their ill-adapted morphology makes large spiders clumsy walking on the ground [38]. If, on the other hand, these large spiders choose to bridge, the short distance they can span in a single bridging event will force them to perform a greater number of bridging events to cover the same distance, as compared to smaller individuals, thus expending more time and energy by having to build more lines [38]. Bridging imposes a final cost on large spiders linked to the GH of SSD [25,31]. The actual trajectory of a spider during bridging has some resemblance with an inverted parabola: from the midpoint on, spiders must actually climb to reach their goal. If climbing speed decreases in spiders of relatively large mass, as a few studies have shown, [25,29,31], large size will be disadvantageous during the final stages of bridging. Even though some studies have failed to find a negative relationship between body size and climbing speed in spider males [26,30], the lack of this pattern in males, which exhibit a small range of variation in size than females, could be the product of the "ghost of the evolution past" [8]. This would imply that we could not find a climbing disadvantage for larger males because the sizes of extant males fall below the threshold beyond which body size constraints climbing speed. Indeed, using a sufficiently wide range of body sizes, researchers showed that the relationship between climbing speed and this trait has an optimum at intermediate value, after which climbing speed decreases with body size, as predicted by the GH [31].

Given that males are the searching sex in spiders [19], selective pressures on morphological traits enhancing mobility in general, and bridging in particular, should be much stronger in males than in females. Hence, in species living in high habitats, where bridging is a common mode of locomotion [32-34,36], being a good "bridger" could be adaptive for males, since they could be favored from sexual selection by scramble competition. Previous studies have already suggested the advantage of more mobile males related to scramble competition in spiders with extreme SSD as well as the implications in the evolution of SSD [25,36,53,54]. Considering the biomechanical constraint of the model mentioned above and the low rate at which giant females seem to bridge, we hypothesise that there should be a negative relationship between body size and bridging ability and that this bridging constraint should have played a role in the evolution of extreme SSD in spiders. In this paper we test these hypotheses.

**Figure 1:**
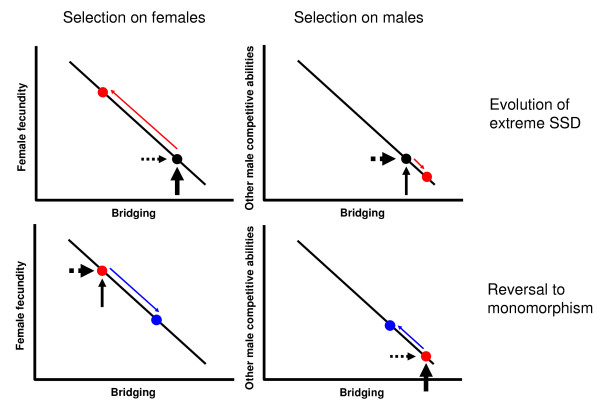
**Size-mediated trade-offs involving bridging ability**. How a size-mediated trade-off between fecundity and bridging ability (females) and a size-mediated trade-off between other male competitive abilities (e.g. walking on the ground, male-male contest competition) and bridging ability (males) can lead to the evolution of extreme SSD and the reversal to monomorphism. Black circles, ancentral bi-variate trait; Red circles, trait values after the response to opposite selection in females and males; Blue circles, values after response to selection converging to similar female and male sizes. The horizontal dotted arrows denote the strength of selective forces (i.e. favouring bridging) decreasing body size and the vertical solid arrows denote the strength of selective forces increasing body size. The red and blue arrows denote the trajectories of the response to selection along the trade-off line. When fecundity selection in females is stronger than selection for dispersal, female gigantism evolves. When selection for dispersal by bridging is stronger in males, male dwarfism evolves. The join effect is the evolution of extreme SSD. Similarly, when dispersal is favoured over fecundity in females and other male competitive abilities are favoured over bridging in males, reversal to monomorphism will evolve.

A more comprehensive way to introduce these predictions is assuming a trade-off between traits positively correlated to body size and traits negatively correlated to body size (Figure 1). Assuming heritability in body size, if bridging is negatively correlated with body size and other traits are positively correlated with large size, such as fecundity in females [7], and in males walking on the ground [26] and/or advantages in male-male contest competition [55], two scenarios are possible, the evolution of extreme SSD and the evolution from extreme SSD to reversed monomorphism [13]. The evolution of male and female body sizes along the trajectory of the trade-off will depend on the direction and magnitude of the net effect of opposing selective forces, those that favor a large body size on the one hand and those that favor a small body size on the other. Furthermore, this net effect of opposing selective forces will also have to overcome the genetic correlation between the sexes [9] for extreme SSD to evolve. Reversal to monomorphism could evolve in environments in which dispersal by bridging is more important in females than fecundity (such as highly unpredictable environments for prey availability) and/or contest competition or walking on the ground is more important than bridging for males (such as habitats with sparse vegetation).

**Figure 2:**
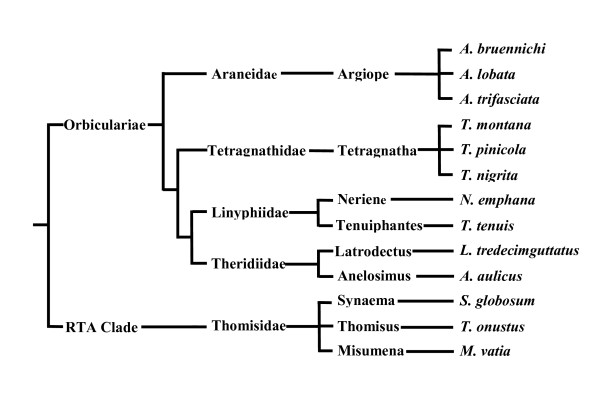
**Phylogeny of the spiders used for the analysis comparing sex differences in bridging propensity to SSD**. This phylogeny was rebuilt using published information for the different taxa.

Here we use bridging propensity, i.e., the probability of building a bridging line and crossing it by walking upside-down under laboratory conditions of constant wind speed, as a proxy for the ease with which spiders can bridge in nature. The rationale of the approach is that if larger spiders have a stronger constraint for bridging, then selection should have favored a lower propensity to disperse by this mechanism in larger individuals. We measured bridging propensity in laboratory conditions for adult males and females of 13 spider species of considerable phylogenetic diversity (Table 1; Figure 2), both within the Orbicularian clade and the family Thomisidae -RTA clade-, covering a broad range of spider body sizes (Table 1). After the trials were finished all the spiders were weighed and their carapace width (CW) measured. To analyze the data we used phylogenetically controlled analyses. We predicted that i) SSD would explain sex differences in bridging propensity, in the sense that when both males and females are small and of similar size both would bridge at the same rate, but when females start growing to a large size and males remain relatively small, then females would bridge at a much lower rate; ii) there is a negative relationship between body size and the probability of bridging for both males and females.

**Table 1:** Average Body Mass and Carapace Width (CW) for males and females of each species (Values are Mean ± SD).

Species	Sex	n	Mass (mg)	CW (mm)	Bridging propensity	SDI mass	SDI CW
*Argiope bruenichi*	female	2	546.500 ± 406.304	4.689 ± 0.544	0	24.718	1.095
	male	2	21.250 ± 0.778	2.238 ± 0.057	1		

*Argiope lobata*	female	21	982.224 ± 406.480	6.112 ± 0.535	0	39.178	1.375
	male	19	24.447 ± 4.166	2.573 ± 0.107	0.842		

*Argiope trifasciata*	female	1	341.500	4.997	0	12.837	1.607
		1	24.680	1.917	1		

*Tetragnatha montana*	female	5	52.140 ± 5.553	2.052 ± 0.100	0	1.427	0.122
	male	5	21.480 ± 4.452	1.830 ± 0.072	1		

*Tetragnatha pinicola*	female	3	33.067 ± 9.139	1.870 ± 0.007	1	0.886	0.131
	male	10	17.530 ± 10.123	1.653 ± 0.143	1		

*Tetragnatha nigrita*	female	1	7.950	1.265	1	-0.094	-0.145
	male	1	8.700	1.449	1		

*Neriene emphana*	female	5	20.280 ± 0.733	1.544 ± 0.054	0.4	4.965	0.173
	male	1	3.400	1.316	1		

*Tenuiphantes tenuis*	female	4	0.748 ± 0.788	0.770 ± 0.051	1	0.459	0.023
	male	11	0.482 ± 0.417	0.753 ± 0.036	1		

*Latrodectus tredecimguttatus*	female	14	522.707 ± 158.910	5.179 ± 0.356	0	27.488	1.872
	male	12	18.348 ± 4.493	1.803 ± 0.181	0.833		

*Anelosimus aulicus*	female	13	4.627 ± 2.500	1.013 ± 0.064	1	0.941	0.015
	male	3	2.383 ± 0.252	0.998 ± 0.019	1		

*Synaema globosum*	female	24	35.098 ± 13.758	2.164 ± 0.250	0.625	5.470	0.267
	male	16	5.425 ± 2.236	1.707 ± 0.124	0.875		

*Thomisus onustus*	female	14	257.025 ± 127.050	3.859 ± 0.415	0.071	59.150	1.513
	male	16	4.003 ± 1.162	1.539 ± 0.105	0.875		

*Misumena vatia*	female	2	151.750 ± 11.809	3.304 ± 0.480	0	59.700	1.263
	male	1	2.500	1.460	1		

Bridging propensity is the proportion of females or males that bridge.

## Results

### Bridging propensity *vs*. SSD

**Figure 3:**
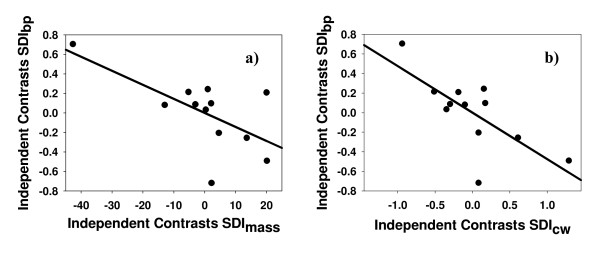
**Sex differences in bridging propensity (SDI_bp_) *vs*. SSD (SDI_SSD_)**. SSD was measured as an SDI index either using body mass (a) or carapace width (b). Also an SDI index was calculated for sex differences in bridging propensity (see text for more details about how the SDI index is built). The plots show the linear relationship between SSD-**a**) SDI_mass_; **b**) SDI_CW_- and sex differences in bridging propensity (SDIbp). Points are independent contrasts.

**Figure 4:**
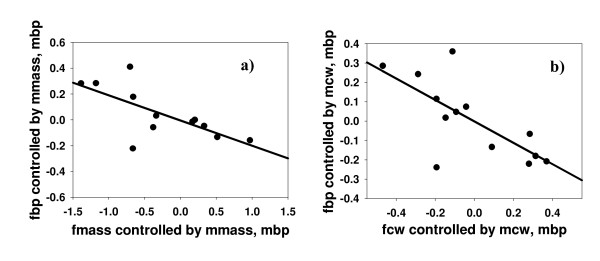
**Multivariate Generalized Least Squares (GLS) approach to test for a relationship between SSD and sex differences in bridging propensity**. **a) **Partial regression plots between female bridging propensity (fbp) in the y axis and female body mass (fmass) in the x axis, controlled for male body mass (mmass) and male bridging propensity (mbp). **b) **carapace width is used instead of body mass; fcw, female carapace width; mcw, male carapace width. All variables were log-transformed (see text for more details).

As predicted, we found a negative relationship between SSD and sexual dimorphism in bridging propensity. The phylogenetically controlled GLS analysis showed that the differences in bridging propensity across species were significantly explained by SSD, whether measured as body size (CW, t_11 _= -3.296, p = 0.004) or as body mass (t_11 _= -2.440, p = 0.016). This relationship was negative for both variables (Figure 3, see also tables A1, A2 in Additional file 1, and Figure A1 in Additional file 2), which implies that when females are much larger than males, males bridge at higher rates relative to females (see Methods below for a more detailed explanation of how the SDI index was built). The variation of the GLS analyses including male bridging propensity as well as female and male body size as independent variables, with female bridging propensity as the dependent variable, also revealed that SSD was negatively related to differential bridging propensity. After controlling for male body size and male bridging propensity, female body size was negatively related to female bridging propensity for both CW and body mass (t_9 _= -3.059, p = 0.007 and t_9 _= -3.631, p = 0.003, respectively; Figure 4, table A3 in Additional file 1). Taken together, our results show that a lower bridging propensity of females is linked to stronger female-biased SSD across taxa.

### Bridging propensity *vs*. size in females and males

**Figure 5:**
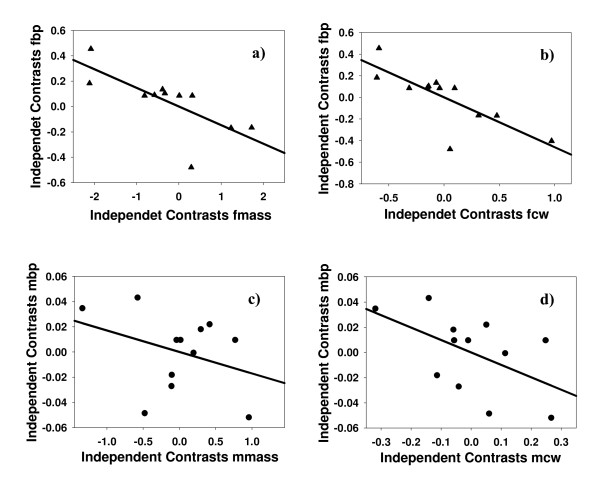
**Relationship between body size and bridging propensity**. Plots show the relationship between either body mass (mass) or carapace width (CW) and bridging propensity (bp) for both sexes. **a) **female bp (fbp) *vs*. body mass (fmass); **b) **female bp (fbp) *vs*. female CW (fcw), **c) **male bp (mbp) *vs*. male body mass (mmass) **d) **male bp (mbp) *vs*. male carapace width (mcw). All variables were log-transformed (see text for more details). Points are independent contrasts.

Both females and males tended to bridge less when larger. The GLS analyses ran separately for females showed that female size, measured either as CW or body mass, explained the proportion of bridging females, with the larger taxa having a lower propensity to bridge (Female mass: t_11 _= -4.751, p < 0.001; Female CW: t_11 _= -4.528, p < 0.001; Figure 5a, b). Likewise, males with wider carapaces were significantly less prone to bridge (Male CW: t_11 _= -2.350, p = 0.019), and a negative relationship was also found for body mass, although this last relationship was only marginally significant (Male mass: t_11 _= -1.668, p = 0.061; Figure 5c, d; see also tables A4, A5 in Additional file 1, and Figure A2 in Additional file 2). The slopes for males and females (tables A4 and A5 in Additional file 1) differ in almost one order of magnitude, being those of males much flatter than those of females. Actually, when we combined males and females in the same GLS analysis we found a significant interaction between sex and body size, measured either as mass (t_22 _= -3.093, p = 0.003) or CW (t_22 _= -2.516, p = 0.020; table A6 in Additional file 1).

### Empirical probability of bridging as a function of size

**Figure 6:**
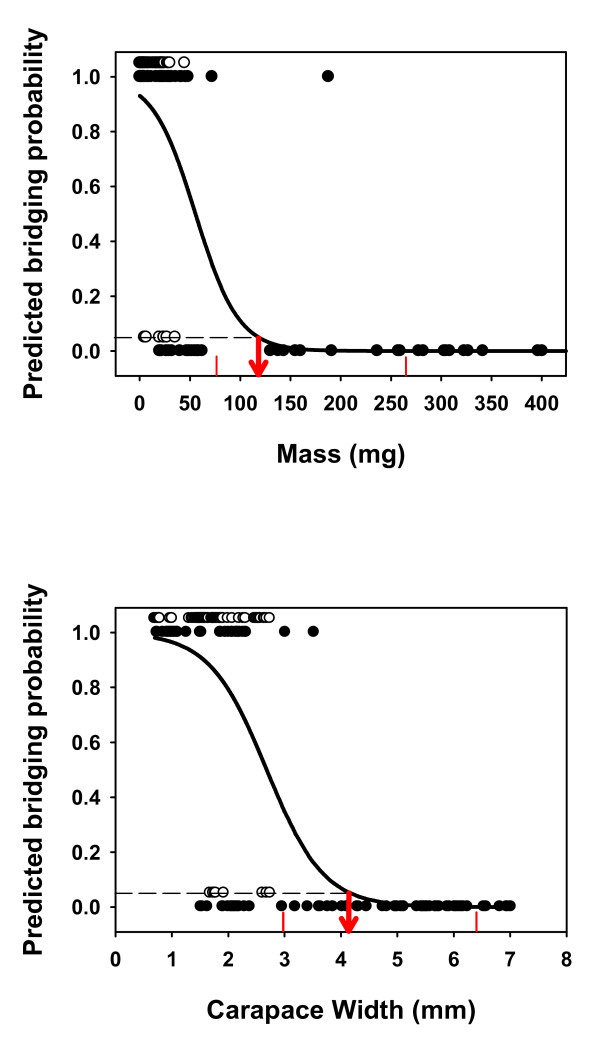
**Effects of spider size on the probability of bridging**. Plots show the probability of bridging predicted by logistic regressions (continuous line) using either **a) **body mass or **b) **carapace width as predictor variables. Each dot corresponds to the trial of one single individual (females solid circles, males empty circles). To distinguish between males and females, successful bridging takes the value of 1 for females and 1.05 for males, while unsuccessful bridging takes the value of 0 for females and 0.05 for males. The red arrows show the threshold beyond which the predicted probability of bridging is les than 5%, and the red small vertical lines mark the limits of the confidence interval for this threshold.

Figure 6 shows the predicted probability of bridging extracted from logistic regressions using body mass (figure 6a) and body size (CW, figure 6b) as predictor variables. The logistic regression equations are(1)

and(2)

respectively, where body mass, *m*, is expressed in mg and carapace width, *CW*, in mm. These equations can be used to determine the maximum body mass and size beyond which spiders do not bridge. We define the bridging threshold as the body mass or size beyond which the probability of bridging declines below 0.05. Setting *P *= 0.05 in equations 1 and 2 and solving for *m *and *CW*, we obtain that spiders in our sample size were very unlikely to bridge if their body mass was greater than 118.32 mg (95% CI: 76.30 - 259.41) or their carapace was wider than 4.17 mm (95% CI: 2.97 - 6.42) (Figure 6).

## Discussion

As we predicted following our extended version of the GH, SSD (measured using CW or body mass) clearly explains the different bridging abilities between males and females, suggesting that in species living in high habitats, selection has favored small size in males via the enhancement of bridging. Due to the genetic correlation in body size between the sexes, this force acting upon males should have been strong enough to overcome the strong fecundity selection acting on females, which is the driving force of female gigantism. Our results show that in species where females are small and of similar size to males (low SSD), both sexes bridge at a similar, and substantially high rate, but when SSD increases and females become much larger than males, these relatively giant females stop using bridging to move, while males keep bridging at a very high rate. This pattern showing that large body size constrains bridging is also found when we analyze the sexes separately. Thus, body size alone can explain a large part of the variation on the proportion of females that bridged across species. Although we found the same trend in males, the results tended to be less clear - probably because the size of all males examined was too low to severely constrain bridging locomotion (see Figure 6) and a high proportion of males bridged even in the largest species. Therefore, within bridging species, the current body size distribution of male body sizes could be the product of the "ghost of the evolution past" [8], which refers to the fact that the evolutionary processes that we can measure today do not necessarily reflect adaptive evolution occurring in the past [see also [27]]. In other words, if we measure natural selection within a species in nature, and find that smaller sizes are not favored during mate search, the underlying reason could be that all male sizes fall below the bridging threshold, and this by no means would mean that selection for bridging had not originated the pattern of SSD in the evolutionary past (see however an example below suggesting bridging selection against large males in natural conditions), but rather that past selection has been stronger in males (see evidence for this below). The strength of the evolutionary comparative method relies precisely in comparing species (or taxa), allowing expanding the range of variation of traits and thus increasing the chances of detecting adaptive patterns. Together, our results confirm a size-mediated trade-off involving bridging which leads to the evolution of extreme SSD. In species living in high habitats, selection would have favored a small size in males via the enhancement of bridging. By contrast, in females, a relatively low environmental stochasticity in prey availability would select for fecund (and thus large) females that would need to disperse relatively little, leading to the evolution of extreme SSD. A reversal to monomorphism could also occur when an increase in environmental stochasticity for prey availability selects for high dispersal rates in females or when male-male contest competition or other dispersal modes favoring a large size become more important for males. The fact that the slope of the relationship between size and bridging propensity is much steeper in females than in males also supports the hypothesis that selection in the past has been much stronger in males than in females, making the slope of the trade-off to tilt up in males [56].

A question remaining to be tested would be whether the evolution of SSD is a cause or a consequence of bridging dispersal in elevated habitats. Although the final answer will have to wait for a larger study than this one, including species with and without SSD, with and without bridging dispersal, our data suggest that selection to preserve bridging in males has induced SSD when female size has increased. At least in the Orbicularian clade, the ancestral state would be small males and females [13], and we can hypothesize that both sexes bridged (certainly the males: males bridge in all Orbicularian species that we have tested). If this was indeed the ancestral state, it follows that through the evolution of the clade females of different species have repeatedly and independently foregone bridging because the advantages of increasing body size (increased fecundity) were larger than the costs of losing mobility. Males, on the other hand, have remained small enough to bridge. In this scenario, SSD is the result of two selective pressures: on females to increase their fecundity [13] and on males to retain their bridging ability. For the family Thomisidae, on the other hand, the lack of a resolved phylogeny and the paucity of our data preclude us from advancing even a preliminary answer to the question. However, the hypothesized trade-off in Figure 1, and the evidence supporting it in this paper, suggests that bridging would have always preceded the evolution of extreme SSD, as this is a plausible scenario in which opposing selection for large size in females and small size in males leads to extreme SSD. It is more difficult to think about a possible reversed scenario, in which extreme SSD would lead to an increase in sex differences of bridging propensity.

The mechanical properties of the silk prevent large spiders from bridging (Rodríguez-Gironés et al. [41]), and the threshold of 118.32 mg observed in our data (beyond which the probability of bridging is less than 5%) is in good agreement with the biomechanical model, which predicts that bridging should be constrained beyond masses of 100 mg. Consequently extreme SSD can be expected in high habitats and in those bridging species in which females are larger than the threshold to bridge efficiently, even if they live just barely above the ground. Beyond the bridging threshold, females can still evolve to be larger (due to the advantages originated from fecundity selection), but males will not. Hence, this bridging constraint and the associated trade-off (Figure 1) would decelerate the rate of male and female body size coevolution, relaxing the genetic correlation of body size between the sexes and leading eventually to the evolution of extreme female-biased SSD.

In addition, there is some evidence suggesting that silk properties also depend on the feeding condition of the spider, as starved spiders produce silk of worse quality [57]. This fact would be particularly relevant to females since they must allocate their nutritional and energetic resources not just to produce silk for bridging, but also in the production of offspring and silk for the egg sac [51]. Thus, beyond a certain body size threshold, this reproductive trade-off could have also favored females that do not use bridging locomotion and save resources to invest in reproduction, particularly when fecundity selection is the main driving force of body size. Males, on the other hand, by being released from fecundity selection, could be favored by having large protein reserves to spin the necessary silk to move around at the price of having the burden of carrying heavier body masses during mate search, which has been shown to be detrimental for bridging [54]. Alternatively, selection could favor a smaller size that would allow males to bridge efficiently without having a high demand of nutrients. Assuming that the cost of energy and nutrient expenditure of smaller males is relatively less than the benefit of bridging more efficiently, we propose that the second explanation is more likely to be true.

The hypothesis that selection favoring bridging efficiency in males has been responsible for the evolution of extreme SSD is not incompatible with other hypotheses that explain selection on small males. On the contrary, we suggest that our hypothesis is complementary. For example, the Differential Mortality Model predicts that smaller males are favored because the high predation risk that males suffer during mate searching relaxes male-male contest competition for females, and ultimately selection favoring large males. This in turn favors early maturation because it improves male viability and his chances to reproduce [15,58]. Certainly, direct selection favoring smaller bridging males and indirect viability selection also favoring smaller males can work synergistically. The GH [25] also predicts that small males are favored because they move faster in vertical surfaces. Additionally, a recent revision of this hypothesis shows a curvilinear relationship between body mass an climbing speed, proposing that extreme SSD would have evolved in species where females live in high habitats and are larger than the optimal body mass for climbing [31]. Whether selection for fast climbing or efficient bridging has been stronger over evolutionary time depends on how often spiders use each of these two kinds of locomotion, and also on how often they walk on the ground, which has actually been shown to favor relatively larger males [26,30]. These different sources of selection need to be evaluated with field work. Nonetheless, as far as we know, there is only one study (using males of *Nephila clavipes*) that has evaluated different kinds of locomotion in spiders, and this study shows that bridging is by far the most frequent mechanism used to move and that smaller males are favored at finding mates [36]. However, we would like to stress that this extended version of the Gravity Hypothesis, also considering bridging locomotion, supports the more global explanation that in general gravity is an important factor to explain the evolution of extreme SSD. Furthermore, this "Bridging GH" even explains the exceptional cases in which there is no extreme SSD in species with relatively giant females living on relatively tall vegetation, as it is the case for some species within the families Theraphosidae, Ctenidae, Oxyopidae and Sparassidae. The absence of extreme SSD in these taxa could be related to the fact that these species have not evolved bridging capabilities or bridging morphologies [38] and consequently the selective pressures to keep males small would be less strong. Finally, our hypothesis and the fecundity selection hypothesis [7] are not mutually exclusive either, as the second only explains why females are large, not why males are relatively small in some species with large females and not in others. Rather, a bridging-fecundity trade-off acting on females can help to explain the evolution of extreme SSD (Figure 1).

## Conclusions

Bridging is a neglected dispersal mode that can explain the evolution of extreme SSD in spiders. Physical constraints make bridging inefficient for large spiders. Thus, in species where bridging is a very common mode of locomotion, small males, by being more efficient bridgers, will enjoy more mating opportunities and thus will be better at scramble competition to reach receptive females. While there is general agreement that fecundity selection increasing female size is quantitatively the most significant factor that can explain the actual pattern of SSD in spiders [7,13,16,17] our hypothesis helps to solve the controversial question of what keeps the males small [5], and also contributes to explain the wide range of SSD in spiders, which the fecundity hypothesis can not explain. Hence, extreme SSD should always be expected in species that commonly use bridging locomotion and in which females are large and have a low need to disperse.

## Methods

### Spider collection

We collected adult male and female spiders in four different areas: Cabo de Gata and Punta Entinas (Almería, South East Spain), Cadí-Moixeró Natural Park (Pyrenees, North East Spain) and Region of Villuercas-Ibores (Extremadura, South West Spain) between May 2006 and May 2007. We selected our samples from the two independent clades in which most of the examples of extreme SSD are found, Orbiculariae and the family Thomisidae within the RTA Clade [13]. Preliminary data from a study including temperate spiders of about 58 genera and 21 families across the entire spider phylogeny (Agelenidae, Amaurobiidae, Dictynidae, Dysderidae, Gnaphosidae, Linyphiidae, Lycosidae, Miturgidae, Oecobiidae, Oxyopidae, Philodromidae, Pholcidae, Pisauridae, Salticidae, Sparassidae, Teraphosidae, Tetragnathidae, Theridiidae, Thomisidae and Titanoecidae) show that bridging locomotion has been detected only in one additional clade to those included here: the Dictynidae (Corcobado & Moya-Laraño *unpublished data*). The fact that there is a considerable overlap between the clades where most cases of extreme SSD are found and those where bridging has been recorder so far, suggests a possible link between the distribution of bridging locomotion and that of extreme SSD across spiders. Within the two selected clades, we chose our sample in order to maximize phylogenetic diversity and to include those taxa with a body shape (relationship between leg lengths vs. body size) that suggested bridging locomotion [38]. In total, the dataset comprised a total of 204 individuals from 13 species: *Argiope bruennichi*, *Argiope lobata *and *Argiope trifasciata *(Araneidae), *Tetragnatha montana*, *Tegragnatha pinicola *and *Tetragnatha nigita*. (Tetragnathidae), *Neriene emphana *and *Tenuiphantes tenuis *(Linyphiidae), *Latrodectus tredecimguttatus *and *Anelosimus aulicus *(Theridiidae), *Synaema globosum*, *Thomisus onustus *and *Misumena vatia *(Thomisidae). Our sample of species covered a wide range of body sizes, including almost the entire range for temperate web-building spiders [59]: Females - Carapace Width (CW) 0.7-7.02 mm, Body Mass 0.2-1846.6 mg; Males - CW 0.7-2.75 mm, Body Mass 0.3-44.8 mg.

### Bridging trials

All spiders were kept in the laboratory in jars of variable size adjusted to their own size until the trials were performed. All trials were performed at room temperature, during the day and within the next 24-72 hours after the spiders had been collected. Temperature during the bridging trials, time of day when the trials were run and time elapsed since the spiders were captured had no significant effect in our response variable and thus we did not include them in further analyses. We followed the methods described in Moya-Laraño et al. [38] to experimentally induce bridging in the laboratory, with minor modifications to adapt the system to our larger range of spider sizes. We placed a blowing fan 3.3 m away from a plant fragment and released the spider on top of a wire stand 27 cm height, which was located between the plant and the fan (30 cm away from the plant and 3 m away from the fan). The fan produced an air flow with a speed of 0.6 - 0.8 m·s^-1 ^blowing on the top of the wire stand. All trials were recorded with a video-camera for as long as 5 minutes. Our response variable was whether the spider bridged or not. If spiders did not start releasing silk for a bridge within the first 2 minutes, we induced spiders to do so by poking them gently a few times with a paint brush. Trials were finished when the spider reached the plant after a successful bridging or 3 minutes after we first poked them, whatever came first. Of the 126 spiders that bridged, 61 needed to be poked before they started bridging. Notice that this allowed us to be sure that all spiders were motivated to bridge if they could do so. Thus, all spiders were likely responding to a potential predatory threat, the difference being that some of them responded immediately upon handling and others needed to be specially "threatened". After trials were finished all the spiders were weighed with a precision balance to the nearest 0.1 mg or 0.01 mg (smaller spiders were weighed with higher precision). The spiders collected in Almería were weighed alive after the trials, and then killed by freezing. The rest of the spiders were frozen after the trials and carried to the laboratory to be weighed as frozen individuals. Previously, we had verified that there was almost no difference between the weight of alive or frozen spiders (Repeatability between measurements - alive vs. frozen: R > 0.99; p < 0.001; n = 28). All spiders were preserved in 70% ETOH after weighing. We measured the size of specimens (CW) under a dissection microscope. All animals were measured by the same observer (GC) with high intra-observer repeatability: R = 0.94; p < 0.001; n = 20.

### Statistical Analyses

#### Measurement of Sexual Size Dimorphism (SSD)

SSD was estimated from CW and body mass. CW is fixed at maturation and does not change with the feeding status of the spider [reviewed in [12]], while body mass is a more dimorphic and plastic character [e.g. [12,60]] that needs to be investigated because some biomechanical properties of the spider silk suggest that body mass is the trait that could constrain the ability to bridge efficiently (Rodríguez-Gironés et al. *unpublished manuscript*). We used the modified ratio index or SDI [61] to measure SSD because of its good statistical properties [10]. This index is estimated by taking the ratio of the larger to the smaller sex minus 1, and then assigning a negative value if males are the larger sex, and a positive value if females are larger. The index takes a value of 0 when there is no SSD.

#### Comparative Analysis

To analyze the data across species we used a type of Generalized Least Squares (GLS) that incorporates phylogenetic relationships to correct for non-independence due to common ancestry [62]. The GLS analysis was implemented in R using the package PHYLOGR [63]. We rebuilt the phylogeny for the species included in our data using published partial phylogenies [13,64-70].

We included polytomies when the available phylogeny was not completely resolved. Because branch length is unknown for most phylogenies we assigned all branches the same arbitrary value of one. Phylogenies were drawn using the PDTREE procedure within the statistical package PDAP [71] and then the phylogenetic distance matrix was imported into PHYLOGR. Figure 2 shows the phylogeny of our sample of species.

To illustrate the results of those analysis that only involved one predictor variable we generated the independent contrasts (IC) following the method of Garland et al. [71] using the package PDAP [71], -the statistical package used here to run the GLS do not provide the possibility to draw scatter plots in which points are corrected for phylogenetic distances-. The Method of IC and the GLS are functionally identical [72], although the former has lower statistical power when the phylogeny includes some politomies (due to a decrease in the degrees of freedom). In addition, GLS has the advantage that it accommodates multiple independent variables. The results of the analyses following the IC methods are included in tables A2 and A5 of Additional file 1.

Because our sample size was very small for some species (See Table 1), we repeated the analysis excluding the four species that had only one individual for either sex. The results were qualitatively the same, with the p-values only marginally significant for some of the analyses due to the smaller sample size (see tables B1, B2, B3 of Additional file 3). Thus, our results are robust despite the relatively small sample size for some of the species used.

#### Testing prediction i): SSD explains the differences in bridging propensity across taxa

In order to asses the robustness of our results we applied two different approaches. First, we calculated the proportion of males and females that bridged for each species, and then we used these data to calculate a modified SDI index of bridging propensity (SDI_bp_). The modification was required because, in some species, the proportion of bridging females was equal to zero, and the SDI index is undefined when the minimal value (the denominator) is zero. To avoid dividing by zero we added one to the numerator and denominator of the quotient of the SDI index. We obtained the average values of CW for each sex and species, and then we calculated SDI using CW (SDI_cw_). Similarly we used the average mass for each sex and species to calculate mass SDI (SDI_mass_). We then ran two GLS analyses using SDI_bp _as the dependent variable and either SDI_cw _or SDI_mass _as predictor variables. We predicted a negative relationship between differential bridging propensity and SSD. Note, that the prediction is a negative relationship between SDIbp and SDIcw or SDImass because SDIs are arbitrarily made negative when males are the larger sex. Thus, when the SDIcw and SDImass are large and positive (large females), the SDIbp is predicted to be large and negative (males bridge more than females). In the second approach we followed the method used by De Mas et al. [58], which is an adaptation of Smith's suggestion for analyzing SSD through multiple regression using log-transformed variables and introducing statistical control [73]. As we did in the previous analysis, we introduced the average for each sex and species in the multiple regression model. Thus, we ran a GLS comparative analysis in which we included female bridging propensity as the dependent variable and female body size, male body size, and male bridging propensity as independent variables. All the variables were log-transformed using natural logarithms, although for bridging propensity we calculated the logarithm of one plus the proportion of bridging individuals (since, as mentioned above, bridging propensity included zeros). We predicted that female body size, statistically controlled in the model for male body size, would show a negative relationship with female bridging propensity, which was statistically controlled for male bridging propensity by including this variable on the right hand side of the model. As the prediction is for a negative relationship, all statistical tests were one-tailed. We used partial regression plots to graphically display the results of the multiple GLS analyses [74].

#### Testing prediction ii): There is a negative relationship between body size and bridging propensity

First, we tested whether body size (CW) or body mass explains the propensity to bridge separately for males and females. We then combined males and females in the same analysis and tested for an interaction between sex and body size. To perform this analysis we add at the tip of the main phylogeny an additional node with two branches, one for each sex (sex was coded as follows: males 0, females 1). For each sex we used the average of either CW or body mass as independent variables and the proportion of bridging individuals as the dependent variable. All the variables were log-transformed using the natural logarithm, but as above, for bridging propensity we added one to the raw value before log-transformation. Again, as the prediction is for a negative relationship, all statistical tests were one-tailed.

To calculate the threshold size above which spiders did not bridge we performed generalized linear models with binomial error and logit link functions using "size" (body mass or CW) as the predictor and occurrence or non- occurrence of bridging as the dependent variable. The predicted regression equations were then used to calculate the size beyond which the probability of bridging was very low (<0.05). To obtain confidence intervals for this threshold size, for each measure of size (body mass or CW), we generated 1000 logistic equations by randomly generating the equation parameters (slope and intercept). For each randomly generated equation, the values of the intercept and the slope were independent realizations of normally distributed random variables, with mean and standard deviation given by the expected value and standard error of the corresponding parameter in the original (data-driven) regression equation. The size (body mass or CW) for which the simulated logistic equation predicted a probability of bridging of 0.05 was stored for each of the 1000 runs, thus providing 1000 estimates of the threshold size. Eliminating the 2.5% highest and lowest values, we obtained the 95% confidence intervals for these parameters. Because we know of no method to include binomial errors in the evolutionary comparative method, this analysis did not include a correction for phylogenetic relatedness and treated individual spiders as independent data points. Although this analysis was not used for hypothesis testing, we realize that not correcting for phylogenetic relationships could substantially affect parameter estimation. However, a recent study using a very similar approach [31] showed that the calculation of optimal climbing speed in spiders changed very little regardless of whether phylogenetic correction was applied or not. Thus, here we assume that parameters would also change little (or at least not meaningfully) if we had applied a phylogenetic correction.

## Authors' contributions

GC and JM-L conceived and designed the study. JM-L supervised the study. All authors captured spiders and performed trials. GC weighed and measured the specimens and ran the statistical analyses. MAR-G performed the analysis on the empirical probability of bridging. JM-L drew figure 1. GC, JM-L and MAR-G wrote the manuscript. All the authors read, made significant comments and approved the final manuscript.

## Supplementary Material

Additional file 1 **Extended statistics tables**. This file includes tables A1-A6 with additional statistical information related to the analyses included in the manuscript. Click here for file

Additional file 2 **Plots of raw data**. This file includes scaterplots of raw data (points are not controlled for philogenetic distance). Figure A1 shows the relationship between SSD and sex differences in bridging propensity; Figure A2 shows the relationship between body size and bridging propensity. Click here for file

Additional file 3 **Additional statistics tables**. This file includes tables B1-B3 with additional statistical information related to the main analyses included in the manuscript, but in which we excluded the 4 species which had only one individual in either sex. Thus, here analyses include only 9 species. Click here for file

## References

[B1] Darwin C The descent of man, and selection in relation to sex 1871 London: John Murray

[B2] Andersson M Sexual Selection 1994 Princeton: Princeton Univ. Press

[B3] Abouheif E Fairbairn DF A comparative analysis of allometry for sexual size dimorphism: assessing Rensch's rule Am Nat 1997 149 540 562 10.1086/286004

[B4] Fairbairn DJ Fairbairn DJ, Blanckenhorn WU, Székely T Introduction: the enigma of sexual size dimorphism Sex, Size & Gender Roles: Evolutionary Studies of Sexual Size Dimorphism 2007 New York: Oxford University Press 1 10

[B5] Blanckenhorn WU The evolution of body size: What keeps organisms small? Q Rev Biol 2000 75 385 407 10.1086/393620 11125698

[B6] Roff DA Life Hystory Evolution 2002 Sunderland: Sinauer Associates

[B7] Head G Selection on Fecundity and Variation in the Degree of Sexual Size Dimorphism Among Spider Species (Class Araneae) Evolution 1995 49 776 781 10.2307/2410330 28565139

[B8] Blanckenhorn WU Behavioral causes and consequences of sexual size dimorphism Ethology 2005 111 977 1016 10.1111/j.1439-0310.2005.01147.x

[B9] Lande R Sexual dimorphism, sexual selection, and adaptation in polygenic characters Evolution 1980 34 292 305 10.2307/2407393 28563426

[B10] Fairbairn DJ Blanckenhorn WU Székely T Sex, size & gender roles: evolutionary studies of sexual size dimorphism 2007 Oxford: Oxford University Press

[B11] Fairbairn DJ Allometry for sexual size dimorphism: Pattern and process in the coevolution of body size in males and females Annu Rev Ecol Syst 1997 28 659 687 10.1146/annurev.ecolsys.28.1.659

[B12] Foellmer MW Moya-Laraño J Fairbairn DJ, Blanckenhorn WU, Szekely T Sexual Size Dimorphism in Spiders Sex, Size & Gender Roles. Evolutionary studies of sexual size dimorphism 2007 New York: Oxford University Press 71 81

[B13] Hormiga G Scharff N Coddington JA The phylogenetic basis of sexual size dimorphism in orb-weaving spiders (Araneae, Orbiculariae) Syst Biol 2000 49 435 462 10.1080/10635159950127330 12116421

[B14] Main BY Dwarf males in mygalomorph spiders: adaptation to environmental hazards Act Zool Fenn 1990 190 273 278

[B15] Vollrath F Parker GA Sexual Dimorphism and Distorted Sex-Ratios in Spiders Nature 1992 360 156 159 10.1038/360156a0

[B16] Coddington JA Hormiga G Scharff N Giant female or dwarf male spiders? Nature 1997 385 687 688 10.1038/385687a0

[B17] Prenter JR Elwood W Montgomery WI Sexual size dimorphism and reproductive investment by female spiders: A comparative analysis Evolution 1999 53 1987 1994 10.2307/2640458 28565440

[B18] Prenter J Elwood RW Montgomery WI Mate guarding, competition and variation in size in male orb-web spiders, *Metellina segmentata*: a field experiment Anim Behav 2003 66 1053 1058 10.1006/anbe.2003.2266

[B19] Foelix RF Biology of Spiders 1996 2 Oxford: Oxford University Press

[B20] Aisenberg A Viera C Costa FG Daring females, devoted males, and reversed sexual size dimorphism in the sand-dwelling spider Allocosa brasiliensis (Araneae, Lycosidae) Behav Ecol Sociobiol 2007 62 29 35 10.1007/s00265-007-0435-x

[B21] Huxley JS Darwin's theory of sexual selection and the data subsumed by it, in the light of recent research Am Nat 1938 72 416 433 10.1086/280795

[B22] Ghiselin M The Economy of nature and the Evolution of Sex 1974 Berkeley: University of California Press

[B23] Elgar MA Sexual Cannibalism, Size Dimorphism, and Courtship Behavior in Orb-Weaving Spiders (Araneidae) Evolution 1991 45 444 448 10.2307/2409679 28567867

[B24] Schneider JM Herberstein ME De Crespigny FC Ramamurthy S Elgar MA Sperm competition and small size advantage for males of the golden orb-web spider *Nephila edulis* J Evol Biol 2000 13 939 946 10.1046/j.1420-9101.2000.00238.x

[B25] Moya-Laraño J Halaj J Wise DH Climbing to reach females: Romeo should be small Evolution 2002 56 420 425 11926508 10.1111/j.0014-3820.2002.tb01351.x

[B26] Brandt Y Andrade MCB Testing the gravity hypothesis of sexual size dimorphism: are small males faster climbers? Funct Ecol 2007 21 379 385 10.1111/j.1365-2435.2007.01243.x

[B27] Moya-Laraño J Vinković D Allard C Foellmer M Gravity still matters Funct Ecol 2007 21 1178 1181 10.1111/j.1365-2435.2007.01335.x

[B28] Brandt Y Andrade MCB What is the matter with the gravity hypothesis? Funct Ecol 2007 21 1182 1183 10.1111/j.1365-2435.2007.01345.x

[B29] Moya-Laraño J Vinković D Allard CM Foellmer MW Mass-mediated sex differences in climbing patterns support the gravity hypothesis of sexual size dimorphism Web Ecol 2007 7 106 112

[B30] Prenter J Perez-Staples D Taylor PW The effects of morphology and substrate diameter on climbing and locomotor performance in male spiders Funct Ecol 2010 24 400 408 10.1111/j.1365-2435.2009.01633.x

[B31] Moya-Laraño J Vinković D Allard CM Foellmer MW Optimal climbing speed explains the evolution of extreme sexual size dimorphism in spiders J Evol Biol 2009 22 954 963 10.1111/j.1420-9101.2009.01707.x 19243487

[B32] Moore CW Life-Cycle, Habitat and Variation in Selected Web Parameters in Spider, *Nephila Clavipes *Koch (Araneidae) Am Midl Nat 1977 98 95 108 10.2307/2424717

[B33] Peters HM On the structure and glandular origin of bridging lines used by spiders for moving to distant places Act Zool Fenn 1990 190 309 314

[B34] Peters HM Kovoor J The Silk-Producing System of Linyphia-Triangularis (Araneae, Linyphiidae) and Some Comparisons with Araneidae - Structure, Histochemistry and Function Zoomorphology 1991 111 1 17 10.1007/BF01632706

[B35] Morse DH Fritz RS Experimental and Observational Studies of Patch Choice at Different Scales by the Crab Spider *Misumena vatia* Ecology 1982 63 172 182 10.2307/1937042

[B36] Linn CD The effect of male size on travel ability in the colden orb-weaving spider *Nephila clavipes*: implications for sexual size dimorphism MSc 2001 Tulane University, Department of Psychology

[B37] Anderson JT Morse DH Pick-up lines: cues used by male crab spiders to find reproductive females Behav Ecol 2001 12 360 366 10.1093/beheco/12.3.360

[B38] Moya-Laraño J Vinković D De Mass E Corcobado G Moreno E Morphological evolution of spiders predicted by pendulum mechanics PLoS ONE 2008 3 e1841 10.1371/journal.pone.0001841 18364999 PMC2266996

[B39] Bonte D De Clercq N Zwertvaegher I Lens L Repeatability of dispersal behaviour in a common dwarf spider: evidence for different mechanisms behind short- and long-distance dispersal Ecol Entomol 2009 34 271 276 10.1111/j.1365-2311.2008.01070.x

[B40] Bonte D Inbreeding depresses short and long distance dispersal in three congeneric spiders J Evol Biol 2009 22 1429 1434 10.1111/j.1420-9101.2009.01756.x 19460080

[B41] Rodríguez-Gironés MA Corcobado G Moya-Laraão J Silk elasticity as a pontential constraint on spider body size J Theor Biol 210 266 430 435 10.1016/j.jtbi.2010.06.031 20600136

[B42] Hayashi CY Blackledge TA Lewis RV Molecular and mechanical characterization of aciniform silk: Uniformity of iterated sequence modules in a novel member of the spider silk fibroin gene family Mol Biol Evol 2004 21 1950 1959 10.1093/molbev/msh204 15240839

[B43] Blackledge TA Swindeman JE Hayashi CY Quasistatic and continuous dynamic characterization of the mechanical properties of silk from the cobweb of the black widow spider *Latrodectus hesperus* J Exp Biol 2005 208 1937 1949 10.1242/jeb.01597 15879074

[B44] Blackledge TA Hayashi CY Silken toolkits: biomechanics of silk fibers spun by the orb web spider *Argiope argentata *(Fabricius 1775) J Exp Biol 2006 209 2452 2461 10.1242/jeb.02275 16788028

[B45] Eberhard WG How Spiders Initiate Airborne Lines J Arachnol 1987 15 1 9

[B46] Vollrath F Kohler T Mechanics of silk produced by loaded Proc R Soc Lond B 1996 263 387 391 10.1098/rspb.1996.0059

[B47] Vollrath F Madsen B Shao ZZ The effect of spinning conditions on the mechanics of a spider's dragline silk Proc R Soc Lond B 2001 268 2339 2346 10.1098/rspb.2001.1590 PMC1088885 11703874

[B48] Garrido MA Elices M Viney C Perez-Rigueiro J Active control of spider silk strength: comparison of drag line spun on vertical and horizontal surfaces Polymer 2002 43 1537 1540 10.1016/S0032-3861(01)00713-3

[B49] Blackledge TA Zevenbergen JM Condition-dependent spider web architecture in the western black widow, *Latrodectus hesperus* Anim Behav 2007 73 855 864 10.1016/j.anbehav.2006.10.014

[B50] Tso IM Chiang SY Blackledge TA Does the giant wood spider Nephila pilipes respond to prey variation by altering web or silk properties? Ethology 2007 113 324 333 10.1111/j.1439-0310.2007.01318.x

[B51] Vollrath F Biology of spider silk Int J Bio Macromol 1999 24 81 88 10.1016/S0141-8130(98)00076-2 10342751

[B52] Lubin Y Ellner S Kotzman M Web relocation and habitat selection in a desert widow spider Ecology 1993 74 1915 1928 10.2307/1940835

[B53] Legrand RS Morse DH Factors driving extreme sexual size dimorphism of a sit-and-wait predator under low density Biol J Linn Soc 2000 71 643 664 10.1111/j.1095-8312.2000.tb01283.x

[B54] Ramos MM Irschick D Christenson T Overcoming an evolutionary conflict: Removal of locomotor performance PNAS 2004 101 4883 4887 10.1073/pnas.0400324101 15034176 PMC387343

[B55] Elgar MA Birkhead TR, MÖller AP Sperm competition and sexual selection in spiders and other arachnids Sperm competition and sexual selection 1998 London: Academic Press 307 332 full_text

[B56] Roff DA Fairbairn DJ The evolution of trade-offs: where are we? J Evol Biol 2007 20 433 447 10.1111/j.1420-9101.2006.01255.x 17305809

[B57] Madsen B Shao ZZ Vollrath F Variability in the mechanical properties of spider silks on three levels: interspecific, intraspecific and intraindividual Int J Bio Macromol 1999 24 301 306 10.1016/S0141-8130(98)00094-4 10342779

[B58] De Mas E Ribera C Moya-Laraño J Resurrecting the differential mortality model of sexual size dimorphism J Evol Biol 2009 22 1739 1749 10.1111/j.1420-9101.2009.01786.x 19627415

[B59] Roberts MJ Spiders of Britain and Northern Europe 1995 London: Harpen Collins

[B60] Prenter J Montgomery WI Elwood RW Multivariate morphometrics and sexual dimorphism in the orb-web spider *Metellina segmentata *(Clerck, 1757) (Araneae, Metidae) Biol J Linn Soc 1995 55 345 354

[B61] Lovich JE Gibbons JW A review of techniques for quantifying sexual size dimorphism Growth Dev Aging 1992 56 269 281 1487365

[B62] Grafen A The phylogenetic regression Phil Trans R Soc Lond B 1989 326 119 157 10.1098/rstb.1989.0106 2575770

[B63] Diaz-Uriarte R Garland T PHYLOGR: Functions for phylogenetically based statistical analyses 2007 http://cran.r-project.org/web/packages/PHYLOGR/index.html Accessed August 11, 2009

[B64] Coddington JA Ubick D, Cushing PE, Paquin PE, Roth V Phylogeny and classification of spiders Spiders of North America: an identification manual American 2005 American Arachnological Society 18 24

[B65] Arnedo MA Coddington JA Agnarsson I Gillespie RG From a comb to a tree: phylogenetic relationships of the comb-footed spiders (Araneae, Theridiidae) inferred from nuclear and mitochondrial genes Mol Phylogenet Evol 2004 31 225 245 10.1016/S1055-7903(03)00261-6 15019622

[B66] Agnarsson I Morphological phylogeny of cobweb spiders and their relatives (Araneae, Araneoidea, Theridiidae) Zool J Linn Soc-Lond 2004 141 447 626 10.1111/j.1096-3642.2004.00120.x

[B67] Benjamin SP Dimitrov D Gillespie RG Hormiga G Family ties: molecular phylogeny of crab spiders (Araneae: Thomisidae) Cladistics 2008 24 708 722 10.1111/j.1096-0031.2008.00202.x

[B68] Miller JA Hormiga G The unbearable lightness of being monophyletic: clade stability and the addition of data: a case study from erigonine spiders (Araneae: Linyphiidae, Erigoninae) Cladistics 2004 20 385 442 10.1111/j.1096-0031.2004.00033.x 34892954

[B69] Alvarez-Padilla F Systematics of the spider genus Metabus O. P.-Cambridge, 1899 (Araneoidea: Tetragnathidae) with additions to the tetragnathid fauna of Chile and comments on the phylogeny of Tetragnathidae Zool J Linn Soc-Lond 2007 151 285 335 10.1111/j.1096-3642.2007.00304.x

[B70] Kuntner M Coddington JA Hormiga G Phylogeny of extant nephilid orb-weaving spiders (Araneae, Nephilidae): testing morphological and ethological homologies Cladistics 2008 24 147 217 10.1111/j.1096-0031.2007.00176.x

[B71] Garland T Harvey PH Ives AR Procedures for the analysis of comparative data using phylogenetically independent contrasts Sys Bio 1992 41 18 32

[B72] Garland T Ives AR Using the Past to Predict the Present: Confidence Intervals for Regression Equations in Phylogenetic Comparative Methods Am Nat 2000 155 346 364 10.1086/303327 10718731

[B73] Smith RJ Statistics of sexual size dimorphism J Hum Evol 1999 36 423 458 10.1006/jhev.1998.0281 10208795

[B74] Moya-Laraño J Corcobado G Plotting partial correlation and regression in ecological studies Web Ecol 2008 8 35 46

