# Supplementary material for: Introducing the refined gravity hypothesis of extreme sexual size dimorphism
Source: BMC Evol Biol. 2010 Aug 3;10:236. doi: 10.1186/1471-2148-10-236 (PMC2924870; doi:10.1186/1471-2148-10-236)
Supplement: Additional file 1 — Extended statistics tables. This file includes tables A1-A6 with additional statistical information related to the analyses included in the manuscript. [file 1471-2148-10-236-S1.PDF]

Table A1: Univariate Generalized Least Squares (GLS) comparative analyses using SDI bridging propensity (SDI_{bp}) as the dependent variable, and SDI mass or SDI carapace width (SDI_{cw}) as independent variables respectively. Bold letters show significant results.

GLS: Dependent variable SDI_{bp}

Variable	Estimate	SE	t-value	df	P*
**SDI mass**	**-0.0137**	**0.0056**	**-2.440**	**11**	**0.0164**
**SDI cw**	**-0.4837**	**0.1468**	**-3.296**	**11**	**0.0036**

*Test is one-tailed

Table A2: Comparative analyses based on the Independet Contrast method, using SDI bridging propensity (SDI_{bp}) as the dependent variable, and SDI mass or SDI carapace width (SDI_{cw}) as independent variables respectively. Bold letters show significant results.

IC: Dependent variable SDI_{bp}

Variable	Estimate	SE	t-value	df	P*
**SDI mass**	**-0.0144**	**0.0054**	**-2.6345**	**8**	**0.0150**
**SDI cw**	**-0.4767**	**0.1455**	**-3.2753**	**8**	**0.0113**

*Test is one-tailed

Table A3: Multivariate Generalized Least Squares (GLS) comparative analyses including as the dependent variable female bridging propensity (FPB): for each species, the number of bridging females divided by the total number of females tested. As predictor variables the first model includes female body mass (FMASS); male body mass (MMASS) and male bridging propensity (MPB): number of bridging males of each species divided by the total number of males tested for that species. In the second model the variables related to SSD are female carapace width (FCW) and male carapace width (MCW). All variables were log-transformed (see text for more details). Bold letters show significant results relevant to the "Bridging GH".

GLS: Dependent variable FBP

	Independent Variable	Estimate	SE	t-value	df	p*
Mass	**FMASS**	**-0.1942**	**0.0535**	**-3.631**	**9**	**0.0027**
	MMASS	0.0873	0.1068	0.818	9	0.2173
	MBP	-1.6545	1.6970	-0.975	9	0.1775
Carapace Width	**FCW**	**-0.5532**	**0.1809**	**-3.059**	**9**	**0.0068**
	MCW	0.0147	0.4045	0.036	9	0.4859
	MBP	-2.4281	1.8957	-1.281	9	0.1162

*Test is one-tailed

Table A4: Univariate Generalized Least Squares (GLS) comparative analyses using bridging propensity as the dependent variable and body size as the independent variable. Analyses were run for males and females separately. a) Univariate regressions including either female body mass (FMASS) or female carapace width (FCW) predicting female bridging propensity (FBP). b) Univariate regressions including either male body mass (MMASS) or male carapace width (MCW) predicting male bridging propensity (MBP). All variables were log-transformed (see text for more details). Bold letters show significant results.

Table 3a) GLS: Dependent variable FBP

Variable	Estimate	SE	t-value	df	P*
**FMASS**	**-0.1432**	**0.0301**	**-4.751**	**11**	**0.0003**
**FCW**	**-0.4631**	**0.1023**	**-4.528**	**11**	**0.0004**

Table 3b) GLS: Dependent variable MBP

Variable	Estimate	SE	t-value	df	P*
MMASS	-0.0184	0.0110	-1.668	11	0.0615
**MCW**	**-0.0826**	**0.0352**	**-2.350**	**11**	**0.0192**

*Test is one-tailed

Table A5: Comparative analyses based on the Independet Contrasts method using bridging propensity as the dependent variable and body size as the independent variable. Analyses were run for males and females separately. a) Univariate regressions including either female body mass (FMASS) or female carapace width (FCW) predicting female bridging propensity (FBP). b) Univariate regressions including either male body mass (MMASS) or male carapace width (MCW) predicting male bridging propensity (MBP). All variables were log-transformed (see text for more details). Bold letters show significant results.

Table A5a) IC: Dependent variable FBP

Variable	Estimate	SE	t-value	df	P*
**FMASS**	**-0.1470**	**0.0351**	**-4.1805**	**8**	**0.0015**
**FCW**	**-0.4610**	**0.1166**	**-3.9546**	**8**	**0.0021**

Table A5b) IC: Dependent variable MBP

Variable	Estimate	SE	t-value	df	P*
MMASS	-0.0170	0.0147	-1.1601	8	0.1397
**MCW**	**-0.0984**	**0.0501**	**-1.9642**	**8**	**0.0425**

*Test is one-tailed

Table A6: Multivariate Generalized Least Squares (GLS) comparative analyses combining both sexes in the same analysis. The dependent variable was bridging propensity (BP): number of bridging individuals of each sex divided by the total number of individuals of that sex for each species. As predictor variables the first model includes body mass (MASS); SEX and the interaction between SEX and MASS. In the second model we use carapace width (CW) instead of mass to measure body size. All variables (except sex) were log-transformed (see text for more details). Bold letters show significant results.

GLS: Dependent variable BP

	Independent Variable	Estimate	SE	t-value	df	p*
Mass	MASS	-0.0420	0.0368	-1.143	22	0.1327
	SEX	0.1034	0.0874	1.184	22	0.1245
	**SEX * MASS**	**-0.0987**	**0.0319**	**-3.093**	**22**	**0.0027**
Carapace Width	CW	-0.1353	0.14931	-0.907	22	0.1872
	SEX	-0.05782	0.06977	-0.829	22	0.2081
	**SEX * CW**	**-0.30148**	**0.11982**	**-2.516**	**22**	**0.0197**

*Test is one-tailed
