# Supplementary material for: Introducing the refined gravity hypothesis of extreme sexual size dimorphism
Source: BMC Evol Biol. 2010 Aug 3;10:236. doi: 10.1186/1471-2148-10-236 (PMC2924870; doi:10.1186/1471-2148-10-236)

FIG A1

Figure A1: **Difference in bridging propensity between sex (SDI_{bp}) *vs.* SSD (SDI_{SSD}).** SSD was measured either using body mass (a) or carapace width (b) using the SDI index. Also an SDI index was calculated for difference in bridging propensity between sexes. Plots show the relationship of SSD (Fig A1a, SDI_{mass};Fig A1b, SDI_{CW}) and difference in bridging propensity (SDIbp). Points are raw data without controlling for the phylogeny (n=13).

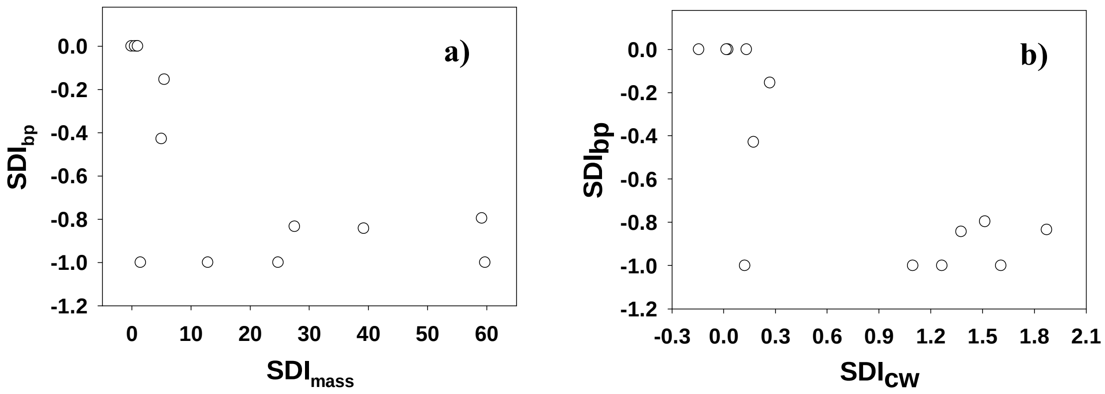

FIG A2

Figure A2: Bridging propensity *vs*. body size.

Plots showing the relationship between body size, measured either as mass (a, c) or as carapace width (cw) (b, d), and bridging propensity (bp). Females (a,b), Males (c,d). All the variables were log-transformed, and in the case of bridging propensity, since the data included 0, we add 1 before log-transformation. Points are raw data without controlling for the phylogeny (n=13)

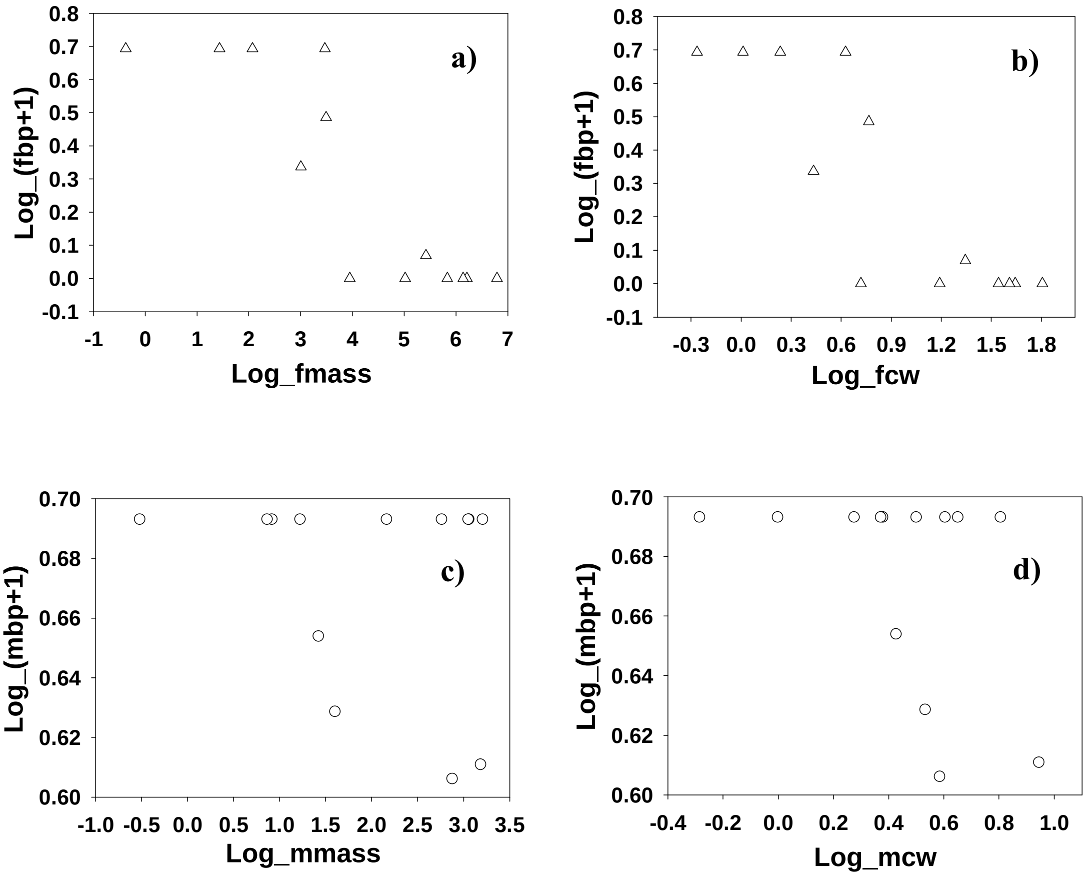

Supplement: Additional file 2 — Plots of raw data. This file includes scaterplots of raw data (points are not controlled for philogenetic distance). Figure A1 shows the relationship between SSD and sex differences in bridging propensity; Figure A2 shows the relationship between body size and bridging propensity. [file 1471-2148-10-236-S2.PDF]
