# Supplementary material for: Introducing the refined gravity hypothesis of extreme sexual size dimorphism
Source: BMC Evol Biol. 2010 Aug 3;10:236. doi: 10.1186/1471-2148-10-236 (PMC2924870; doi:10.1186/1471-2148-10-236)
Supplement: Additional file 3 — Additional statistics tables. This file includes tables B1-B3 with additional statistical information related to the main analyses included in the manuscript, but in which we excluded the 4 species which had only one individual in either sex. Thus, here analyses include only 9 species. [file 1471-2148-10-236-S3.PDF]

Table B1: Univariate Generalized Least Squares (GLS) comparative analyses using SDI bridging propensity ($SDI_{bp}$) as the dependent variable, and SDI mass or SDI carapace width ($SDI_{cw}$) as independent variables respectively. Only 9 species included (those with more than 1 individual of each sex). Bold letters show significant results.

**GLS: Dependent variable $SDI_{bp}$**

| **Variable** | **Estimate** | **SE** | **t-value** | **df** | **P*** |
|---|---|---|---|---|---|
| **SDI mass** | **-0.0147** | **0.0070** | **-2.085** | **7** | **0.0378** |
| **SDI cw** | **-0.4503** | **0.1685** | **-2.672** | **7** | **0.0160** |

*Test is one-tailed

Table B2: Multivariate Generalized Least Squares (GLS) comparative analyses including as the dependent variable female bridging propensity (FPB): for each species, the number of bridging females divided by the total number of females tested. As predictor variables the first model includes female body mass (FMASS); male body mass (MMASS) and male bridging propensity (MPB): number of bridging males of each species divided by the total number of males tested for that species. In the second model the variables related to SSD are female carapace width (FCW) and male carapace width (MCW). All variables were log-transformed (see text for more details). Only 9 species were included (those with more than 1 individual for each sex). Bold letters show significant results relevant to the "Bridging GH".

**GLS: Dependent variable FBP**

| | Independent Variable | Estimate | SE | t-value | df | p* |
|---|---|---|---|---|---|---|
| Mass | **FMASS** | **0.1854** | **0.0960** | **-1.931** | **7** | **0.0555** |
| | MMASS | 0.0924 | 0.1692 | 0.546 | 7 | 0.3040 |
| | MBP | -1.2838 | 2.8769 | -0.446 | 7 | 0.337 |
| Carapace Width | **FCW** | **-0.5354** | **0.2933** | **-1.826** | **7** | **0.0635** |
| | MCW | 0.0985 | 0.5890 | 0.167 | 7 | 0.4370 |
| | MBP | -1.9127 | 3.0768 | -0.622 | 7 | 0.2805 |

*Test is one-tailed

Table B3: Univariate Generalized Least Squares (GLS) comparative analyses using bridging propensity as the dependent variable and body size as the independent variable. Analyses were run for males and females separately. a) Univariate regressions including either female body mass (FMASS) or female carapace width (FCW) predicting female bridging propensity (FBP). b) Univariate regressions including either male body mass (MMASS) or male carapace width (MCW) predicting male bridging propensity (MBP). All variables were log-transformed (see text for more details). Only 9 species were included (those with more than 1 individual for each sex). Bold letters show significant results.

**Table B3a) GLS: Dependent variable FBP**

| Variable | Estimate | SE | t-value | df | P* |
|---|---|---|---|---|---|
| **FMASS** | **-0.1288** | **0.0355** | **-3.630** | **7** | **0.0042** |
| **FCW** | **-0.4183** | **0.1163** | **-3.597** | **7** | **0.0044** |

**Table B3b) GLS: Dependent variable MBP**

| Variable | Estimate | SE | t-value | df | P* |
|---|---|---|---|---|---|
| MMASS | -0.0179 | 0.0114 | -1.573 | 7 | 0.0800 |
| **MCW** | **-0.0788** | **0.0368** | **-2.14** | **7** | **0.0348** |

*Test is one-tailed
